# Prognostic Impact of the Signet Ring Cell Type in Node-Negative Gastric Cancer

**DOI:** 10.1038/srep26313

**Published:** 2016-07-06

**Authors:** Pengfei Kong, Ruiyan Wu, Chenlu Yang, Qirong Geng, Jianjun Liu, Shangxiang Chen, Xuechao Liu, Minting Ye, Wenzhuo He, Qiong Yang, Liangping Xia, Dazhi Xu

**Affiliations:** 1 State Key Laboratory of Oncology in South China, Collaborative Innovation Centre for Cancer Medicine, Guangzhou, China; 2 Department of the VIP region, Sun Yat-sen University Cancer Centre, Guangzhou, China; 3 Department of the Gynaecologic Oncology Surgery, Sun Yat-sen University Cancer Centre, Guangzhou, China; 4 Department of Hematology Oncology, Sun Yat-sen University Cancer Center, Guangzhou, China; 5 Department of Gastric and Pancreatic Surgery, Sun Yat-sen University Cancer Centre, Guangzhou, China

## Abstract

Little is known regarding the prognostic impact of the signet ring cell (SRC) histotype on negative lymph nodes (LNs) in gastric cancer (GC). In this study, we aimed to investigate the differences between SRC and non-SRC GC patients without LN metastasis. The medical records of patients with GC who underwent gastrectomy at Sun Yat-Sen University Cancer Centre from 1996 to 2012 were reviewed to analyse the clinicopathologic characteristics associated with survival. A total of 480 cases of GC patients without LN metastasis were identified, which included 90 SRC GC patients and 390 non-SRC GC patients. Between the two groups, there were a host of significant differences in the American Joint Committee on Cancer, 7th edition (AJCC) stage. We found that SRC histology was correlated with a poor prognosis in terms of recurrence in node-negative GC patients and that SRC histologic analysis combined with AJCC staging maybe an effectual method for prediction of the recurrence rate. Additionally, we found that SRC GC presents a more dismal overall prognosis in patients with perineural or vascular invasion.

Gastric cancer (GC) is the fourth most common malignancy in the world^1^. The prognostic outcome of GC patients remains ominous, with a 5-year overall survival rate of 25% or less^2^. Nodal metastases represent a known prognostic factor after surgical resection of GC. It is widely accepted that the survival rate improves with a standardized pattern of surgical resection combined with D2 lymphadenectomy^3^,^4^. In theory, patients with negative lymph nodes (LNs) have a good outcome. Nevertheless, approximately 15% of patients with node-negative disease die as a result of postoperative recurrence and metastasis^5^,^6^,^7^.

Since the 1980s, adenocarcinoma with signet ring cell (SRC) features has been reported to be a poor prognostic marker in GC^8^,^9^,^10^. Although the incidence of GC has decreased, there has been a steady increase in the SRC subtype^11^. Thus, the SRC type has been suspected to be a key causative factor of recurrence and metastasis in GC patients. However, its prognostic significance in GC patients with negative LNs remains uncertain. The aim of the current study was to evaluate the long-term effect of the SRC type on the prognosis of LN-negative patients.

## Results

### Patient characteristics and tumour presentation

**Figure 1:**
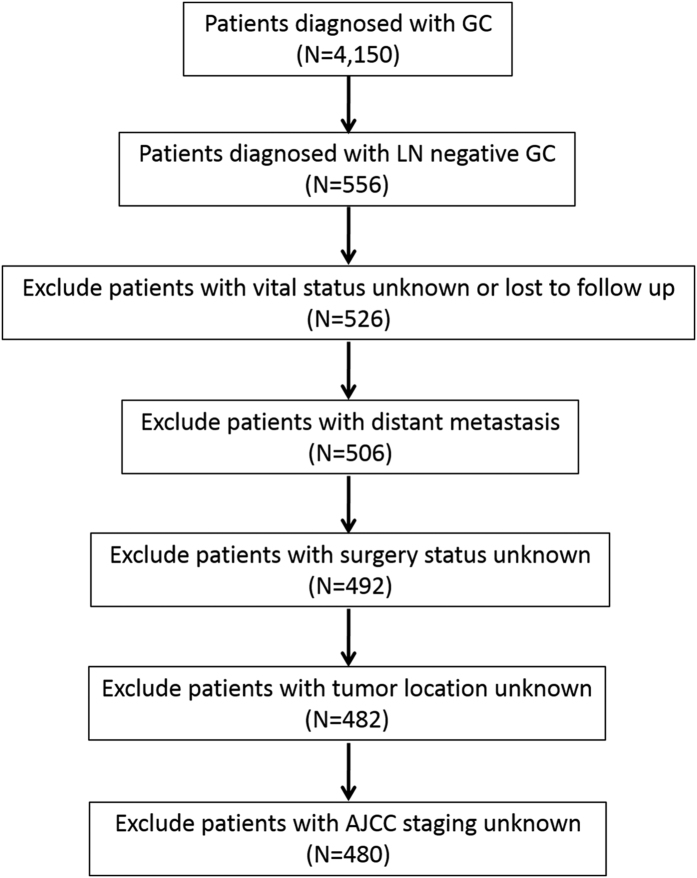
Flow diagram of patient selection. GC, gastric cancer. LN, lymph node. AJCC, American Joint Committee on Cancer, 7th edition (526 gastric cancer patients were included in the flow diagram).

The patient characteristics, tumour presentation, and patient selection are listed in Table 1 and (Fig. 1) (and detailed in the Methods section). The mean follow-up period was 6.2 ± 4.3 years. Of the 480 patients identified for this study, 90 patients (18.8%) had SRC GC, and 390 patients (81.2%) had non-SRC GC. Above all, age, gender, the distribution of location, the tumour grade, the number of retrieved LNs, perineural invasion, vascular invasion and the status of chemotherapy did not show differences between the two groups. SRC presented more commonly at a younger age (57.1 vs. 57.7 years; *P* = 0.646). Both groups were predominantly male, although a smaller proportion of SRC GC patients were male (71.1% vs. 73.1%; *P* = 0.696). The distribution of the anatomic location of the two cancer types is also listed in Table 1 and Supplemental Table 1, based on which we found that a lower proportion of SRC tumours were located in the proximal region of the stomach (22.2% vs. 33.6%; *P* = 0.044). Patients with SRC GC were more likely to present with American Joint Committee on Cancer, 7th edition (AJCC) stage 2 (32.2% vs. 21.8%; *P* = 0.040). However, a lower proportion of patients with SRC GC presented with AJCC stage 1 (53.3% vs. 70.5%; *P* = 0.003). Compared with patients in the non-SRC group, the patients in the SRC group were more commonly in T_2_ (30.0% vs. 16.4%; *P* = 0.004) or T_3_ (32.2% vs. 21.8%; *P* = 0.040). Of note, our results also showed that fewer patients with SRC GC tended to present with T1a (17.8% vs. 47.4%; *P* < 0.001).

All characteristics in which differences were observed were included in our multivariable models. We also compared the characteristics of individuals comprising those excluded from this sample using a multivariable model (Supplemental Table 2). The outcomes indicated that there were no differences between the two groups based on gender distribution, age, or tumour location. Thus, we concluded that our analytic sample broadly represented the demographic and clinical presentation characteristics of all identified LN-negative GC cases, with the above caveats.

### Survival (overall survival and relapse-free survival)

**Figure 2:**
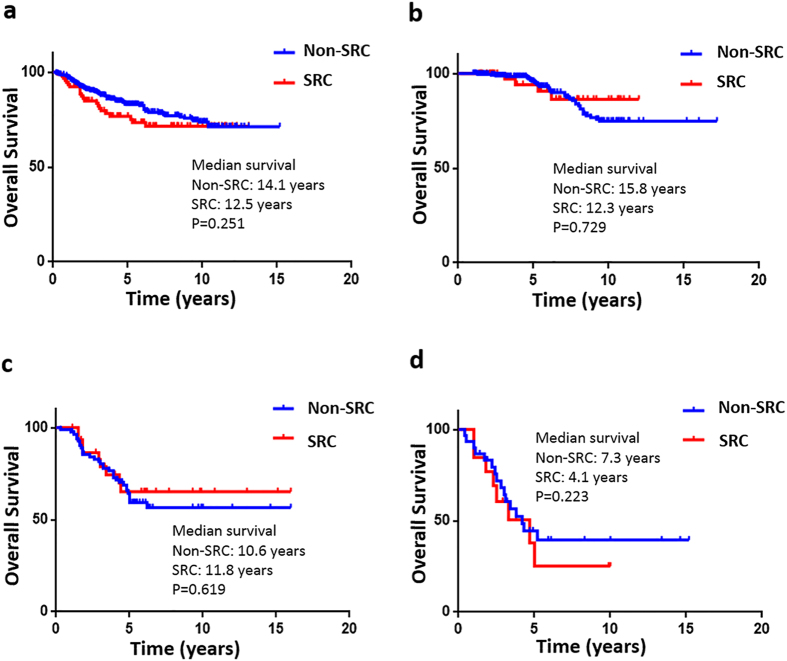
Kaplan-Meier survival curves comparing years of overall survival in signet ring cell (SRC) and non-SRC gastric carcinoma are shown for (**a**) all stages (non-SRC: 380 v SRC: 90), (**b**) American Joint Committee on Cancer, 7th edition (AJCC) stage 1 tumours (non-SRC: 275 v SRC: 48), (**c**) AJCC stage 2 tumours (non-SRC: 85 v SRC: 29), and (**d**) AJCC stage 3 tumours (non-SRC: 30 v SRC: 13).

**Figure 3:**
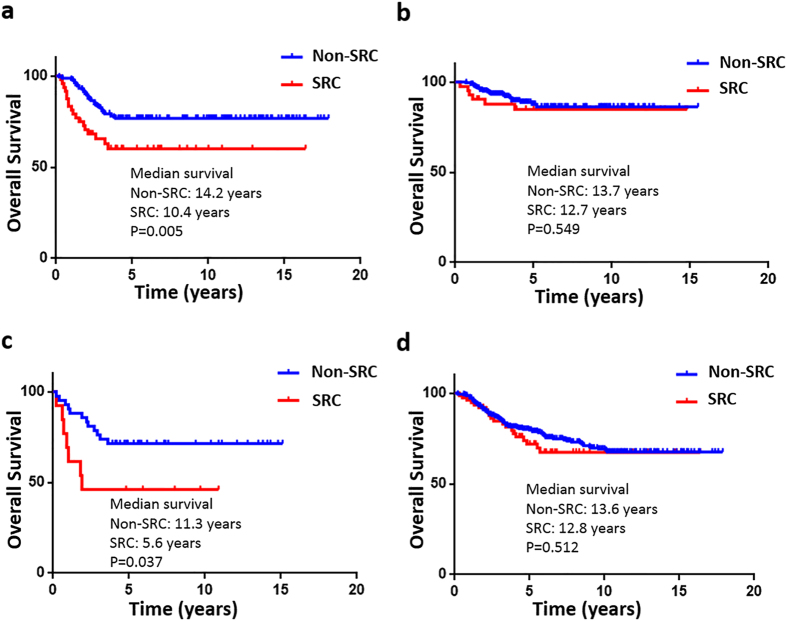
Kaplan-Meier survival curves comparing the years of overall survival in signet ring cell (SRC) and non-SRC gastric carcinoma are shown for (**a**) perineural invasion-positive tumours (non-SRC: 164 v SRC: 48), (**b**) perineural invasion-negative tumours (non-SRC: 226 v SRC: 42), (**c**) vascular invasion-positive tumours (non-SRC: 42 v SRC: 13), and (**d**) vascular invasion-negative tumours (non-SRC: 348 v SRC: 77).

Kaplan-Meier overall survival curves are shown in (Figs 2 and 3). Of the 390 patients with non-SRC GC, 76 patients (19.5%) died. Of the 90 patients with SRC GC, 22 patients (24.4%) died. Overall survival for all AJCC stages in the SRC and non-SRC groups of patients was not significantly different (SRC, 12.5 years vs. non-SRC, 14.1 years; *P* = 0.251). However, a divergence in the overall survival indicated by the Kaplan-Meier curves seemed to emerge before 10 years of follow-up. When comparing stage 1 SRC GC with stage 1 non-SRC GC, survival was not significantly different (12.3 vs. 15.8 years; *P* = 0.729). Similarly, survival for SRC compared with non-SRC GC was also not significantly different for stage 2 (11.8 vs. 10.6 years; *P* = 0.619) or stage 3 (4.1 vs. 7.3 years; *P* = 0.223) (Fig. 2). To determine whether the SRC type has other prognostic relevance, all patients positive and negative for perineural or vascular invasion with known clinical records and long-term follow-up were analysed. In all of these analyses, the results suggest that the SRC type had an obvious independent influence on prognosis in perineural (10.4 vs. 14.2 years; *P* = 0.005) and vascular (5.6 vs. 11.3 years; *P* = 0.037) invasion-positive disease (Fig. 3).

**Figure 4:**
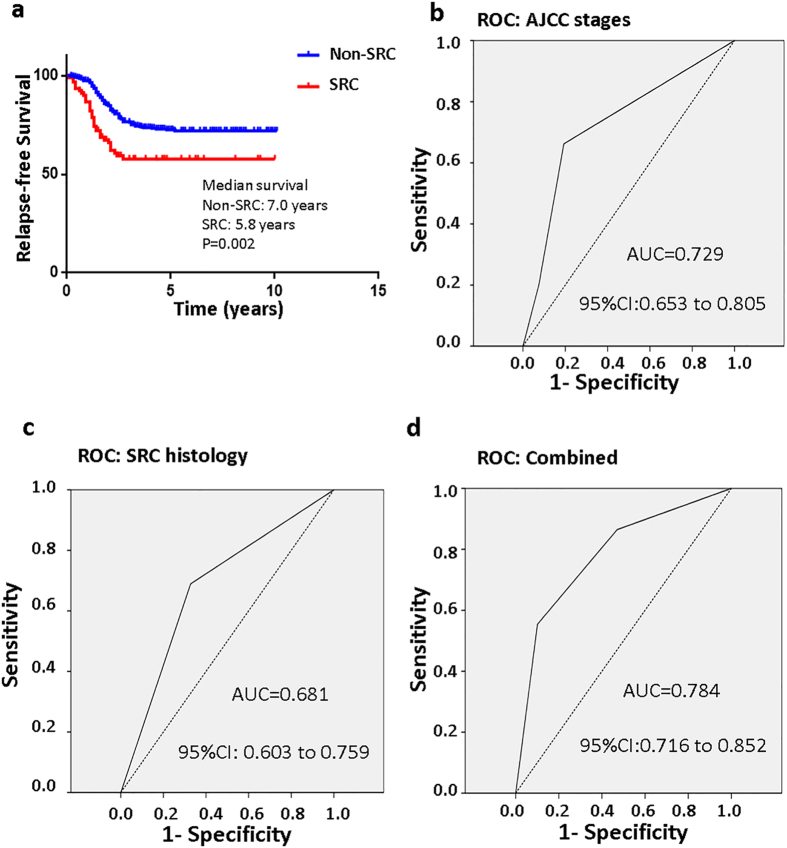
Kaplan-Meier survival curves comparing the years of relapse-free survival in gastric cancer patients. (**a**) Relapse-free survival in signet ring cell (SRC) and non-SRC gastric carcinoma (non-SRC: 380 v SRC: 90). The receiver operating characteristic (ROC) curves for predicting patient relapse-free survival using the American Joint Committee on Cancer, 7th edition (AJCC) stage (**b**), SRC histology (**c**) or a combination of the two factors (**d**). The area under the curve (AUC) and the corresponding 95% confidence interval (CI) are shown in the plots.

Kaplan-Meier curves for relapse-free survival are shown in Fig. 4a. Of all 480 GC patients, 148 had a GC relapse. The prognosis of SRC GC, with median relapse-free survival of 5.3 years, was significantly worse compared with non-SRC adenocarcinomas, with median relapse-free survival of 7.0 years (*P* < 0.001). Moreover, the patterns of recurrence were different between the two groups. Patients with SRC GC had less frequent late recurrence but more frequent early recurrence (*P* = 0.032) (Supplemental Tables 3 and 4).

### Predictors of mortality and recurrence rate

Unadjusted (univariate) associations with mortality are listed in Table 2. The SRC type (univariate Cox hazard ratio (HR), 1.34; 95% confidence interval (CI), 0.83 to 2.15; *P* = 0.232), female sex (univariate Cox HR, 1.25; 95% CI, 0.99 to 1.58; *P* = 0.059), vascular invasion (univariate Cox HR, 1.49; 95% CI, 0.92 to 2.44; *P* = 0.108), the number of retrieved LNs (univariate Cox HR, 1.78; 95% CI, 0.72 to 4.42; *P* = 0.207), the status of chemotherapy (univariate Cox HR, 1.39; 95% CI, 0.97 to 2.00; *P* = 0.071), and the distribution of the anatomic location of cancers added no additional risk as mortality risk factors. Factors associated with increased mortality included the age at diagnosis (univariate Cox HR, 1.04; 95% CI, 1.03 to 1.06; *P* < 0.0001; per year increase in the age at diagnosis), perineural invasion (univariate Cox HR, 3.64; 95% CI, 2.04 to 5.96; *P* < 0.001), increasing AJCC stage, increasing tumour grade, and increasing tumour stage.

The results of the univariate analyses for factors associated with relapse-free survival in node-negative GC are summarized in Table 3. SRC histology (univariate Cox HR, 1.83; 95% CI, 1.23 to 2.70; *P* = 0.003), age (univariate Cox HR, 1.03; 95% CI, 1.01 to 1.04; P < 0.001), perineural invasion (univariate Cox HR, 1.85; 95% CI, 1.32 to 2.60; P < 0.001), vascular invasion (univariate Cox HR, 2.84; 95% CI, 1.91 to 4.22; P < 0.001), advanced tumour grade, and increasing AJCC and tumour stages were significantly associated with relapse-free survival. Moreover, the number of LNs (univariate Cox HR, 2.23; 95% CI, 0.98 to 5.07; *P* = 0.055) tended to be associated with relapse-free survival. In the current study, gender and tumour location were not associated with relapse-free survival.

**Figure 5:**
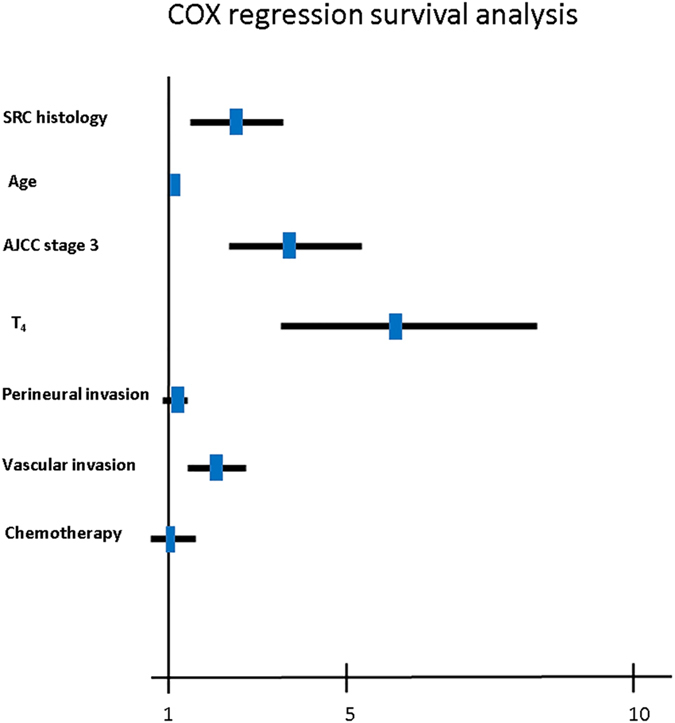
Different factors, including signet ring cell (SRC) carcinoma histology; age; perineural invasion; vascular invasion; American Joint Committee on Cancer, 7th edition (AJCC) stage 3; T_4_; and chemotherapy, were analysed for their association with relapse-free survival using the Cox regression model. The hazard ratio and 95% confidence interval (CI) are plotted for each factor.

To determine the independent prognostic factors associated with overall and relapse-free survival in patients with node-negative GC, probable factors in the multivariate analysis were input into a Cox proportional hazards model (Supplemental Tables 5 and 6). The age at diagnosis (multivariable Cox HR, 1.03; 95% CI, 1.01 to 1.04; *P* = 0.006), female sex (multivariable Cox HR, 1.65; 95% CI, 1.04 to 3.09; *P* = 0.022), perineural invasion (multivariable Cox HR, 1.64; 95% CI, 1.14 to 2.36; *P* = 0.008), and increasing AJCC and tumour stages (Supplemental Table 5) were independently associated with mortality. SRC histology (multivariable Cox HR, 1.13; 95% CI, 0.70 to 1.85; *P* = 0.612) and vascular invasion (univariate Cox HR, 1.32; 95% CI, 0.84 to 2.08; *P* = 0.230) were not found to be independent predictors of mortality. Interestingly, SRC histology (multivariable Cox HR, 2.37; 95% CI, 1.42 to 4.26; *P* = 0.033), age (multivariable Cox HR, 1.03; 95% CI, 1.02 to 1.05; *P* < 0.001), vascular invasion (multivariable Cox HR, 2.16; 95% CI, 1.41 to 3.30; *P* < 0.001) and increasing AJCC and tumour stages (Supplemental Table 6) were significantly associated with relapse-free survival. Therefore, we determined the receiver operating characteristic curves for the prediction of relapse-free survival in node-negative GC patients using the AJCC stage, SRC histology, or a combination of both (Fig. 4b–d). The area under the curve (AUC) for the AJCC staging-based prediction (0.729) was higher than that for the SRC histology-based model (0.681), and the combination of both factors achieved the highest AUC value (0.784). Moreover, multivariate Cox regression survival analysis adjusting for SRC histology, age, AJCC stage 3, T_4_, and vascular invasion consistently validated strong correlations with shorter relapse-free survival in patients, as is also clearly shown in (Fig. 5).

## Discussion

In this study, we examined the effect of SRC histology in LN-negative GC patients on survival and tumour presentation. Our study first demonstrated that SRC patients have worse recurrence and survival rates, yet SRC histology was not an independent prognostic factor for overall survival in LN-negative GC patients. Additionally, another important finding in this study strongly suggests that SRC histology is an independent prognostic factor associated with overall survival in patients with perineural or vascular invasion.

In recent decades, the subset of LN-negative patients who still present a poor prognosis has been the subject of considerable research and controversy^12^,^13^,^14^. Previously, we found that the number of LNs is an independent prognostic factor in LN-negative GC patients. Moreover, we demonstrated that patients with negative LNs who undergo D2 dissection should have at least 16 LNs retrieved for accurate staging^15^. A result consistent with this was also reported by Baiocchi *et al*. in a multicentre study^16^.

Currently, although understanding of the prognostic implications of SRC histology in GC is limited and somewhat conflicting, the majority of studies still show that SRC variants are frequently related to a more aggressive clinical course, especially in Eastern patients^17^,^18^. Therefore, a history of SRC histology might be another factor that contributes to the minority of LN-negative GC patients having poor overall survival.

The primary findings of our study indicate that SRC histology has a correlation with poor prognosis due to recurrence in node-negative GC patients and that SRC histologic analysis combined with AJCC staging maybe an effective method for predicting the recurrence rate of node-negative GC patients. To our knowledge, SRC tumours are a variant of diffuse carcinoma according to Lauren’s recent classification^19^,^20^. To exclude the impact of diffuse carcinoma, we first compared the diffuse type with the non-diffuse type in our enrolled patients, and we subsequently found that the diffuse carcinoma type was not an independent prognostic variable in GC patients with negative LNs (Supplemental Table 7, Supplemental Figs 1 and 2). Furthermore, we found that SRC histology was associated with a poorer overall prognosis in patients with perineural and vascular invasion, which is consistent with the results of other studies on oesophageal cancer and advanced GC patients^21^,^22^. There were several potential reasons for our novel findings. First, SRC GC presents with a more aggressive microscopic appearance that is characterized by abundant intracytoplasmic mucin that pushes the nucleus to the periphery, similar to a signet ring^23^. Second, SRC tumours are considered to be biologically distinct from well-differentiated tumours, which themselves differ according to tumour location and the degree of differentiation^24^. Third, patients with perineural and vascular invasion are usually diagnosed at advanced stages due to late clinical manifestations. The poorer prognosis of GC with SRC histology might be more related to the stage at diagnosis than to histology, but further evidence is needed. For these reasons, SRC GC patients may need more aggressive treatments, such as neoadjuvant or adjuvant chemotherapy, rather than surgery alone. A recent study proposed that preoperative chemotherapy followed by surgery might be a more appropriate treatment for SRC-containing adenocarcinomas of the oesophagogastric junction and stomach^25^.

The secondary finding of our study is that SRC histology was not independently associated with mortality compared with non-SRC histology in LN-negative GC patients. Overall survival did not differ between the two tumour types, though there was a distinct divergence in the overall survival Kaplan-Meier curves presenting before 10 years of follow-up. This finding suggested that overall survival favoured the non-SRC group and that a difference in survival might become evident if more patients are included in future studies. When adjusted for the AJCC stage, the analysis showed that patients with SRC GC had better survival than those with non-SRC GC (12.3 vs. 15.8 years) in AJCC stage 1. However, this increase in survival by 3.5 years was not statistically significant. In addition, the difference in survival between the two groups was not statistically significant in stage 2 or stage 3 GC patients. Several previous studies have reported improved survival with early stages of SRC GC compared with non-SRC GC and relatively worse survival in later stages of the disease^21^,^22^. Among all patients in the present study, 275 patients (70.5%) were stage 1 in the non-SRC group, and 48 patients (53.3%) were stage 1 in the SRC group; the submucosal invasion rates were 52.6% and 83.3%, respectively. Thus, most patients were not in an advanced GC stage, which might explain the rise in the unconformity trend. We did not completely analyse the later stages of GC because we excluded LN-positive patients. In addition, the different results in our study might indicate that the SRC subtype behaves differently in patients in China. Such differences in outcomes could reflect the aggressive screening and resection strategies employed in our country.

Interestingly, we also confirmed that SRC GC has a distinct presentation compared with non-SRC GC, presenting in patients at a more advanced stage. This is in accordance with previous studies conducted in Asian countries and other areas^25^,^26^. According to earlier publications, the two subtypes appear to present at different anatomic locations, with the SRC subtype being more likely to present in the body or the lower stomach^27^. This trend was also observed in the present study, but the difference was not statistically significant. It has been reported that SRC GC patients are younger in age and more likely to be women and present higher tumour grades compared with other types of GC^27^,^28^, but we did not observe these characteristics in the current study. This discrepancy might have been due to the exclusion of patients with LN metastasis, who often present with deeper tumour invasion and a worse prognosis^29^.

These differences in the presentation of SRC GC completely support the emerging concept that SRC GC might actually be a disease distinct from other types of GC, and numerous studies have clearly described this distinction^30^,^31^,^32^. Beyond the results reported here, the histology of SRC can be distinguished based on genetic expression data. Certain researchers have suggested that SRC GC may even be a completely different entity^33^. Furthermore, Sheng *et al*. reported that different molecular subtypes of GC had different responses to PI3-kinase inhibitors and 5-fluorouracil^34^.

There are certain limitations to our study. Notably, an important bias is that SRC features were present in 19.2% of patients in our database, which was significantly higher than the published rate (approximately 10% in all GC patients)^35^. In addition, we must consider that we did not compare different histologic types but rather the SRC type and the non-SRC type. For this reason, we might have overlooked different behaviours depending on the histologic type. Future research on this topic could explore other clearly defined gastric subtypes.

In conclusion, our study’s results are currently the best evidence showing that SRC histology is an independent predictor of poor prognosis due to recurrence in LN-negative GC patients and that SRC histology is also an independent predictor of overall prognosis in patients with perineural and vascular invasion.

## Methods and Patients

### Data source and study sample

The Gastric Cancer database of the Sun Yat-sen University Cancer Centre (SYSUCC), which contains clinicopathologic data from more than 4,000 GC patients, was searched, and 556 patients and 107 patients were identified as having non-metastatic LNs and SRC gastric carcinoma, respectively, as confirmed by postoperative pathologic assessment. These patients all underwent gastrectomy and at least D2 lymphadenectomies for GC, and most of the patients presented a retrieved number of nodes greater than 15 (Supplemental Fig. 3).

Further assessment was performed using the following exclusion criteria: unknown vital status and loss of follow-up data (n = 30), distant metastasis (n = 20), inadequate surgical data (n = 14), unspecified tumour location (n = 10) and unspecified staging (n = 2). As a result, the final group comprised 480 included patients.

All enrolled patients routinely underwent an endoscopic ultrasound or electronic gastroscopy and abdominal computed tomography (CT) examination before surgery to identify the invaded region. After pathologic examination, the diagnosis of GC and the pathologic type were confirmed. The patients underwent routine preoperative examination, including a chest x-ray, an abdominal ultrasound, and blood testing. All of the results before surgery showed no distant metastases and no cancer directly invading the spleen, liver, pancreas, or colon. Thus, all patients underwent radical gastrectomies and at least D2 lymphadenectomies. In our study, Billroth I and II, Roux-en-Y, Roux-en-Y, and jejunal interposition reconstruction were the most common methods of reconstruction after gastrectomy. Additionally, 23 (4.8%) and 112 (23.3%) patients received neoadjuvant and adjuvant chemotherapy, respectively. All specimens were examined by surgeons and pathologists shortly after surgery, and we reviewed all of the resected specimens at this time (Supplemental Fig. 4). According to the World Health Organization criteria for the histologic typing of GC, we defined SRC GC as an adenocarcinoma in which a predominant component (greater than 50% of the tumour) consisted of isolated or small groups of malignant cells containing intracytoplasmic mucin^36^. The status of the LNs was evaluated by pathologists using haematoxylin-eosin staining.

Follow-up data were primarily obtained from the institutional databases, which were regularly updated based on clinical chart reviews, tumour registry information, physician records, patient correspondence, and telephone interviews. Moreover, this study was approved by the Sun Yat-sen University Cancer Centre review board in accordance with Chinese bioethical regulations, and all enrolled patients signed informed consent forms.

### Statistical analysis

To examine unadjusted (univariate) associations, t tests and χ^2^ tests were used. Survival was determined based on cause-specific mortality. Survival curves were generated using the Kaplan-Meier method and were compared using the log-rank test. A Cox proportional hazards model was utilized for multivariate analysis, which included the age at diagnosis, gender, the type of surgical resection and the AJCC TNM staging. Stage was the stratified variable. The data were analysed using SPSS 19.0 statistical software. P < 0.05 was considered statistically significant.

## Additional Information

**How to cite this article**: Kong, P. *et al*. Prognostic Impact of the Signet Ring Cell Type in Node-Negative Gastric Cancer. *Sci. Rep.* **6**, 26313; doi: 10.1038/srep26313 (2016).

## Supplementary Material

Supplementary Table

Supplementary Information

## Figures and Tables

**Table 1:** Comparison of clinicopathologic characteristics between LN-negative SRC and non-SRC GC patients.

Variable	SRC		Non-SRC		*P*
	No. ofPatients(n = 90)	%	No. ofPatients(n = 390)	%	
Age, years					
Mean	57.1		57.7		0.646
SD	10.6		11.7		
Sex					
Male	64	71.1	285	73.1	0.696
Female	26	28.9	105	26.9	
Tumour location					
Upper stomach	20	22.2	131	33.6	0.141
Middle stomach	28	31.1	103	26.4	
Lower stomach	28	31.1	116	29.7	
Overlapping	14	15.6	40	10.3	
AJCC stage					
1	48	53.3	275	70.5	0.006
2	29	32.2	85	21.8	
3	13	14.5	30	7.7	
Tumour grade					
1	12	13.3	74	19.0	0.161
2	19	21.1	103	26.4	
3	59	65.6	213	54.6	
Tumour stage					
T_1a_	16	17.8	185	47.4	<0.001
T_1b_	5	5.6	26	6.7	
T_2_	27	30.0	64	16.4	
T_3_	29	32.2	85	21.8	
T_4_	13	14.4	30	7.7	
Perineural invasion					
Negative	42	46.7	226	57.9	0.060
Positive	48	53.3	164	42.1	
Vascular invasion					
Negative	77	85.6	348	89.2	0.358
Positive	13	14.4	42	10.8	
Number of retrieved LNs					
<15	9	10.0	21	5.4	0.143
≥15	81	90.0	369	94.6	
Chemotherapy					
Yes	29	32.2	106	27.2	0.363
No	61	67.8	284	72.8	

Abbreviations: SRC, Signet Ring Cell. SD, Standard Deviation. AJCC, American Joint Committee on Cancer, 7th Edition. LNs, Lymph Nodes.

**Table 2:** Univariate analyses of prognostic factors for overall survival.

Characteristic	Hazard Ratio	95% CI	*P*
SRC histology	1.34	0.83 to 2.15	0.232
Age at diagnosis	1.04	1.03 to 1.06	<0.001
Female sex	1.25	0.99 to 1.58	0.059
AJCC stage			
1	1.00		<0.001
2	2.89	1.92 to 4.34	
3	4.23	2.78 to 6.42	
Tumour grade			
1	1.00		0.007
2	1.12	0.66 to 4.74	
3	2.25	1.46 to 4.96	
Tumour stage			
T_1a_	1.00		<0.001
T_1b_	0.78	0.41 to 1.47	
T_2_	2.33	1.52 to 3.56	
T_3_	2.79	1.30 to 6.01	
T_4_	4.69	3.03 to 7.26	
Tumour location			
Upper stomach	1.00		0.425
Middle stomach	0.85	0.54 to 1.36	
Lower stomach	0.94	0.60 to 1.46	
Overlapping	1.28	0.67 to 2.91	
Perineural invasion	3.64	2.04 to 5.96	<0.001
Vascular invasion	1.49	0.92 to 2.44	0.108
Number of retrieved LNs	1.78	0.72 to 4.42	0.207
Chemotherapy	1.39	0.97 to 2.00	0.071

Abbreviations: SRC, Signet Ring Cell. AJCC, American Joint Committee on Cancer, 7th Edition. LNs, Lymph Nodes.

**Table 3:** Univariate analyses of prognostic factors for relapse-free survival.

Characteristic	Hazard Ratio	95% CI	*P*
SRC histology	1.83	1.23 to 2.70	0.003
Age at diagnosis	1.03	1.01 to 1.04	<0.001
Female sex	1.21	0.83 to 1.78	0.323
AJCC stage			
1	1.00		<0.001
2	4.10	2.78 to 6.03	
3	9.77	8.23 to 12.04	
Tumour grade			
1	1.00		0.002
2	1.18	0.82 to 1.71	
3	2.89	1.60 to 5.20	
Tumour stage			
T_1a_	1.00		<0.001
T_1b_	1.74	0.99 to 3.07	
T_2_	3.33	2.22 to 4.98	
T_3_	3.51	1.58 to 7.82	
T_4_	5.81	3.84 to 8.78	
Tumour location			
Upper stomach	1.00		0.294
Middle stomach	0.56	0.28 to 1.27	
Lower stomach	1.32	0.88 to 1.99	
Overlapping	1.05	0.71 to 1.56	
Perineural invasion	1.85	1.32 to 2.60	<0.001
Vascular invasion	2.84	1.91 to 4.22	<0.001
Number of retrieved LNs	2.23	0.98 to 5.07	0.055
Chemotherapy	2.49	1.78 to 3.48	<0.001

Abbreviations: SRC, Signet Ring Cell. AJCC, American Joint Committee on Cancer, 7th Edition. LNs, Lymph Nodes.
