# Supplementary material for: Prognostic Impact of the Signet Ring Cell Type in Node-Negative Gastric Cancer
Source: Sci Rep. 2016 Jul 6;6:26313. doi: 10.1038/srep26313 (PMC4933873; doi:10.1038/srep26313)
Supplement: Supplementary Information [file srep26313-s2.pdf]

Supplementary Information

Prognostic Impact of Signet Ring Cell Type in Node Negative Gastric Cancer

Pengfei Kong[1,4],Ruiyan Wu[1],Chenlu Yang[1,3],Jianjun Liu[1,2],Shangxiang Chen[1,2], Xuechao Liu[1,2], Minting Ye[1,4], Min Ma[1,2], Lin Jiao[1], Xuan Li[1], Wenzhuo He[1,4], Qiong Yang[1,4], Chang Jiang[1,4], Fangxin Liao[1,4], Youqing Zhan[1,2], Zhiwei Zhou[1,2], Liangping Xia*[1,4], Dazhi Xu*[1,2]

1 State Key Laboratory of Oncology in South China, Collaborative Innovation Center for Cancer Medicine, Guangzhou, China, 2 Department of Gastric and Pancreatic Surgery, Sun Yat-sen University Cancer Center, Guangzhou, China.3 Department of the Gynecologic Oncology surgery, Sun Yat-sen University Cancer Center, Guangzhou, China. 4Department of the VIP region, Sun Yat-sen University Cancer Center, Guangzhou, China.

**Supplemental Table 1.** Additional comparison of clinicopathologic characteristics between SRC and non-SRC LNs negative gastric cancer patients.
**Supplemental Table 2.** Comparison of clinicopathologic characteristics between excluded and enrolled LNs negative gastric cancer patients.
**Supplemental Table 3.** First site and the timing of recurrence (n = 98).
**Supplemental Table 4.** Total first site and the timing of recurrence (n = 138).
**Supplemental Table 5.** Multivariate analyses of prognostic factors for overall survival.
**Supplemental Table 6.** Multivariate analyses of prognostic factors for relapse-free survival.
**Supplemental Table 7**. Comparison of clinicopathologic characteristics between diffuse and non-diffuse LNs negative gastric cancer patients.
**Supplemental Figure 1.** Kaplan-Meier survival curves comparing years of overall survival between diffuse and non-diffuse LNs negative gastric cancer patients.
**Supplemental Figure 2.** Kaplan-Meier survival curves comparing years of relapse-free survival between diffuse and non-diffuse LNs negative gastric cancer patients.
**Supplemental Figure 3.** Distribution of the number of lymph nodes examined for the entire cohort of 556 patients.
**Supplemental Figure 4.** Hematoxylin and eosin stain of different histologic type gastric carcinoma.

Variable	SRC		Non-SRC		P
	No. of Patients (n=90)	%	No. of Patients (n=390)	%	
Tumor location					
Upper stomach	20	22.2	131	33.6	0.044
Non-upper stomach	70	77.8	259	66.4	
AJCC stage					
2	29	32.2	85	21.8	0.040
1 + 3	61	67.8	305	78.2	
AJCC stage					
1	48	53.3	275	70.5	0.003
2 + 3	42	46.7	115	29.5	
Tumor stage					
T_2	27	30.0	64	16.4	0.004
Non-T_2	63	70.0	326	83.6	
Tumor stage					
T_3	29	32.2	85	21.8	0.040
Non-T_3	61	67.8	305	78.2	
Tumor stage					
T_{1a}	16	17.8	185	47.4	<0.001
Non-T_{1a}	74	82.2	205	52.6	

Abbreviation: SRC, Signet Ring Cell Carcinoma. AJCC, American Joint Committee on Cancer, 7th edition.

Supplemental Table 1. Additional comparison of clinicopathologic characteristics between SRC and non-SRC LNs negative gastric cancer patients.

Variable	Excluded		Enrolled		P
	No. of Patients (n=76)	%	No. of Patients (n=480)	%	
Age, years					
Mean	57		56.2		0.442
SD	11.6		12.3		
Sex					0.687
Men	55	72.4	333	69.4	
Women	21	27.6	147	30.6	
Tumor location					0.601
Upper stomach	31	40.8	221	46.0	
Middle stomach	23	30.2	111	23.1	
Lower stomach	17	22.3	114	23.8	
Overlapping	5	6.6	34	7.1	
AJCC stage					0.347
1	45	59.2	321	66.9	
2	21	27.6	116	24.2	
3	10	13.2	43	8.9	
Tumor grade					0.221
1	32	42.1	216	45	
2	23	30.3	172	35.8	
3	21	27.6	92	19.2	
Tumor stage					0.129
T_{1a}	22	28.9	201	41.9	
T_{1b}	8	10.5	31	6.5	
T_2	18	23.7	91	19.0	
T_3	17	22.4	114	23.8	
T_4	11	14.5	43	8.9	
Perineural invasion					0.263
Negative	48	63.2	268	55.8	
Positive	28	36.8	212	44.2	
Vascular invasion					0.186
Negative	63	82.9	425	88.5	
Positive	13	17.1	55	11.5	
Number of retrieved LNs					0.110
<15	4	5.3	10	2.1	
≥15	72	94.7	470	97.9	
Chemotherapy					0.585
Yes	24		135		
No	52		345		

Abbreviation: AJCC, American Joint Committee on Cancer. 7th edition, LNs Lymph Nodes.

Supplemental Table 2. Comparison of clinicopathologic characteristics between excluded and enrolled LNs negative gastric cancer patients.

	SRC No. of Patients (n=37)			Non-SRC No. of Patients (n=71)			P
	Early	Late	Total	Early	Late	Total	
Liver	14	1	15	15	5	20	0.207
Locoregional	3	1	4	1	3	4	0.486
Peritoneum	2	1	3	8	1	9	0.455
Distant LNs	6	0	6	12	4	16	0.541
Mixed type	8	1	9	14	8	22	0.220
Total	33	4	37	50	21	71	0.032

Early: within 2 years after surgery (≤2 years). Late: 4 years later after surgery (≥4 years). Mixed type: liver or Peritoneum or locoregional and distant lymph node metastasis. LNs: lymph nodes

Supplemental Table 3. First site and the timing of recurrence (n = 108).

	SRC No. of Patients (n=49)				Non-SRC No. of Patients (n=99)			
	Early	Late	Middle	Total	Early	Late	Middle	Total
Liver	14	1	6	21	15	5	15	35
Locoregional	3	1	3	7	1	3	3	7
Peritoneum	2	1	1	4	8	1	2	11
Distant LNs	6	0	1	7	12	4	4	20
Mixed type	8	1	1	10	14	8	4	26
Total	33	4	12	49	48	21	28	99

Early: within 2 years after surgery (≤2 years). Late: 4 years later after surgery (≥4 years). Middle: Between 2 and 4 years. Mixed type: liver or Peritoneum or locoregional and distant lymph node metastasis. LNs: lymph nodes.

Supplemental Table 4. Total first site and the timing of recurrence (n = 148).

Characteristic	Hazard Ratio	95% CI	P
SRC histology	1.13	0.70 to 1.85	0.612
Age at diagnosis	1.03	1.01 to 1.04	0.006
Female sex	1.65	1.04 to 3.09	0.022
AJCC stage			
1	1.00		<0.001
2	2.64	1.64 to 4.24	
3	3.70	2.40 to 5.72	
Tumor stage			
T_{1a}	1.00		<0.001
T_3	2.15	1.21 to 3.16	
T_4	3.09	1.54 to 6.19	
Perineural invasion	1.64	1.14 to 2.36	0.008
Vascular invasion	1.32	0.84 to 2.08	0.230
Chemotherapy	1.77	1.14 to 2.74	0.011

Abbreviation: SRC, Signet Ring Cell Carcinoma. AJCC, American Joint Committee on Cancer, 7th edition.

Supplemental Table 5. Multivariate analyses of prognostic factors for overall survival.

Characteristic	Hazard Ratio	95% CI	P
SRC histology	2.37	1.42 to 4.26	0.033
Age at diagnosis	1.03	1.02 to 1.05	<0.001
AJCC stage			
1	1.00		<0.001
2	2.04	1.12 to 4.26	
3	3.89	2.59 to 5.84	
Tumor stage			
T_{1a}	1.00		<0.001
T_3	2.88	2.04 to 4.21	
T_4	5.34	3.63 to 8.48	<0.001
Perineural invasion	1.39	0.98 to 1.98	0.067
Vascular invasion	2.16	1.41 to 3.30	<0.001
Chemotherapy	1.05	0.70 to 1.59	0.802

Abbreviation: SRC, Signet Ring Cell Carcinoma. AJCC, American Joint Committee on Cancer, 7th edition.

Supplemental Table 6. Multivariate analyses of prognostic factors for relapse-free survival.

Variable	Diffuse		Non-dffuse		P
	No. of Patients (n=167)	%	No. of Patients (n=269)	%	
Age, years					
Mean	56.8		57.7		0.448
SD	12.3		11.0		
Sex					
Men	122	73.1	194	72.1	0.832
Women	45	26.9	75	27.9	
Tumor location					
Upper stomach	57	34.1	80	29.7	0.716
Middle stomach	44	26.3	74	27.5	
Lower stomach	50	29.9	82	30.5	
Overlapping	16	9.6	33	12.3	
AJCC stage					
1	107	64.1	183	68.0	0.112
2	39	23.3	68	25.3	
3	21	12.6	18	6.7	
Number of retrieved LNs					
<15	11	6.6	16	5.9	0.839
≥15	156	93.4	253	94.1	
Chemotherapy					
Yes	52	31.1	71	26.4	0.325
No	115	68.9	198	73.6	

Abbreviation: SRC, Signet Ring Cell Carcinoma. SD, Standard Deviation. AJCC, American Joint Committee on Cancer, 7th edition, LNs, Lymph Nodes.

Supplemental Table 7. Comparison of clinicopathologic characteristics between diffuse and non-diffuse LNs negative gastric cancer patients.

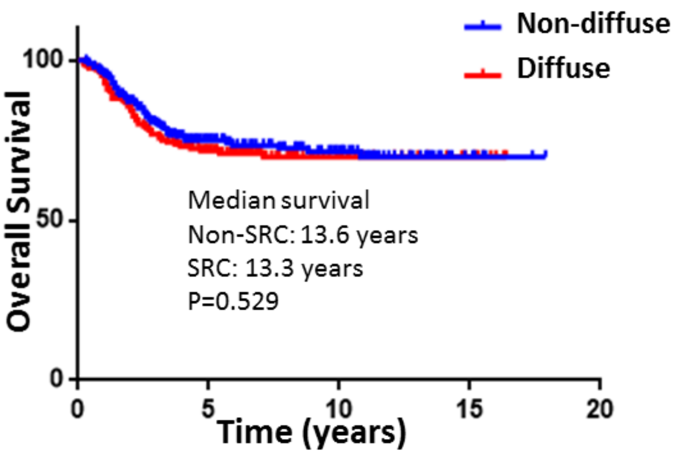

Supplemental Figure 1. Kaplan-Meier survival curves comparing years of overall survival between diffuse and non-diffuse LNs negative gastric cancer patients.

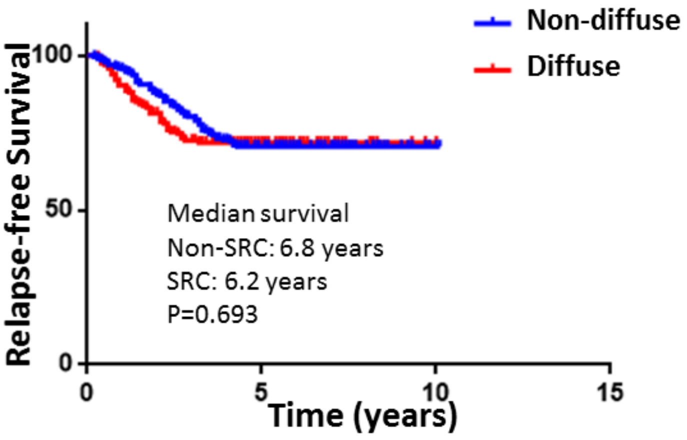

Supplemental Figure 2. Kaplan-Meier survival curves comparing years of relapse-free survival between diffuse and non-diffuse LNs negative gastric cancer patients.

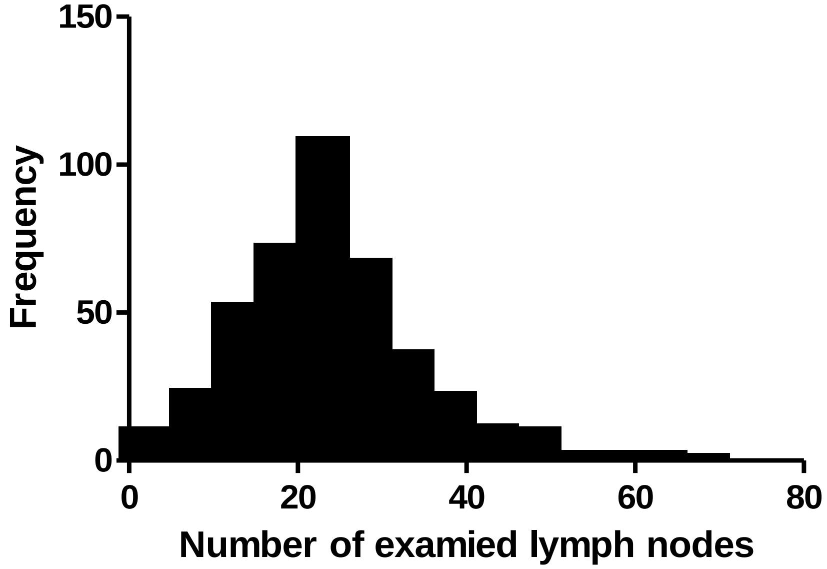

Supplemental Figure 3. Distribution of the number of lymph nodes examined for the entire cohort of 556 patients.

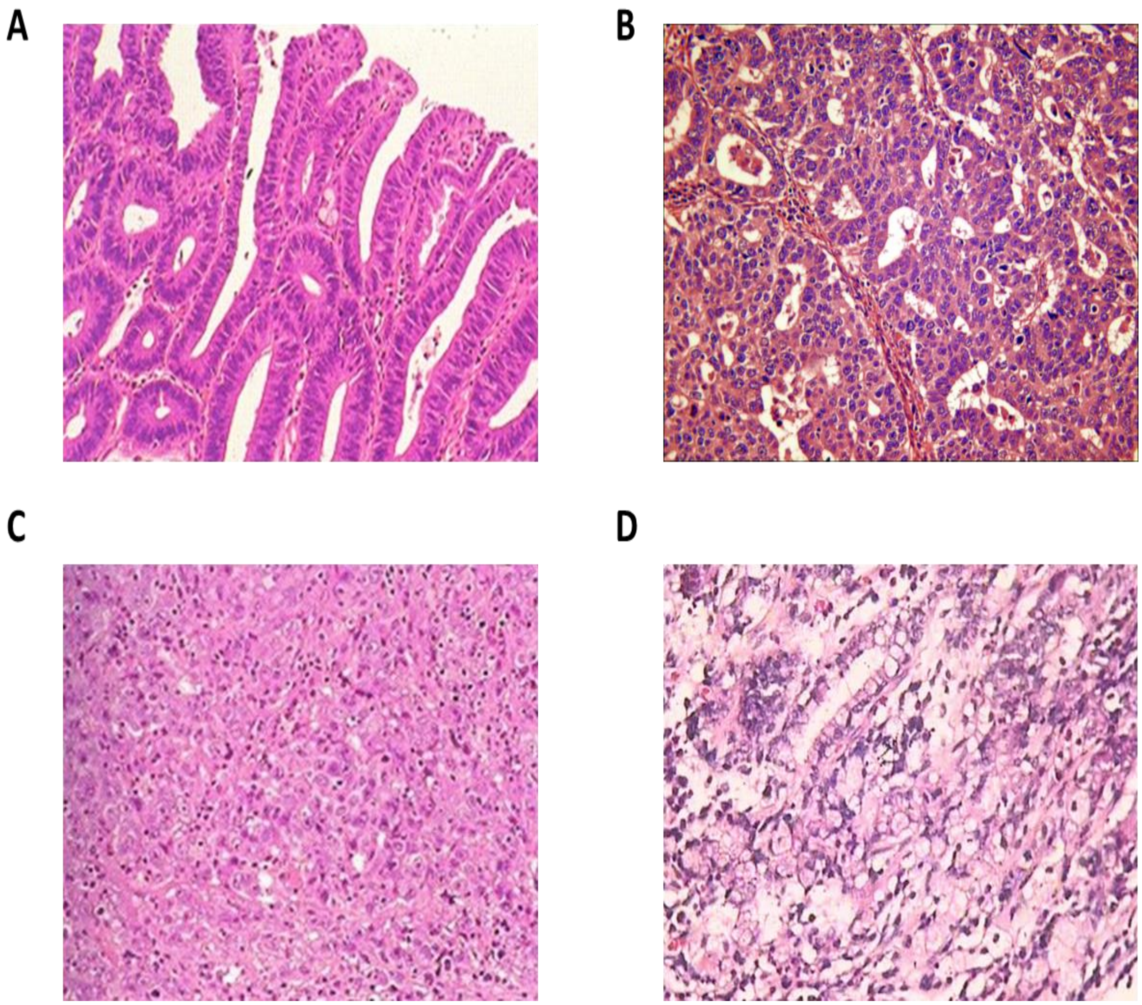

Supplemental Figure 4. Hematoxylin and eosin stain of different histologic type gastric carcinoma. (A) Grade 1 gastric carcinoma, (B) Grade 2 gastric carcinoma, (C) Grade 3 gastric carcinoma, (D) Signet ring cell gastric carcinoma.
